# A multicenter quasi-experimental study: impact of a central line infection control program using auditing and performance feedback in five Belgian intensive care units

**DOI:** 10.1186/2047-2994-2-33

**Published:** 2013-12-05

**Authors:** Soraya Cherifi, Michele Gerard, Sylvie Arias, Baudouin Byl

**Affiliations:** 1 Infection Control Unit, Brugmann University Hospital, 4 Place Van Gehuchten, 1020 Brussels, Belgium; 2 Infection Control Unit, Saint Pierre University Hospital, 322 Rue Haute, 1000 Brussels, Belgium; 3 Infection Control Unit, Erasme University Hospital, 808 Route de Lennik, 1070 Brussels, Belgium; 4 School of Public Health, Université Libre de Bruxelles, Brussels, Belgium

**Keywords:** Audit, Feedback, Intensive care unit, Central-line associated bloodstream infection

## Abstract

**Background:**

We analyzed the impact associated with an intervention based on process control and performance feedback to decrease central line-associated bloodstream infection (CLABSI) rates.

This study was conducted from March 2011 to September 2012 in five adult intensive care units (ICU) located in two Belgian tertiary hospitals A and B, with a total of 53 beds.

**Methods:**

This study was divided in three phases: P1 (baseline), P2 (intervention) and P3 (post intervention).

During P2, external monitoring of five central venous catheters (CVC) care critical processes and monthly reporting (meetings and feedbacks reports posted) of performance indicators (CLABSI rate, CVC utilization ratio, compliance rate with each care process, and insertion site) to ICU workers were performed. The external monitoring of process measures was assessed by the same trained research nurse.

A Poisson regression analysis was used to compare CLABSI incidence density rate per phase. Statistical significance was achieved with 2-sided *p-*value of *<*0.05. For the analysis, we separated the five ICU in hospital A and B when appropriate.

**Results:**

Significantly improved total mean compliance was achieved for hand hygiene, CVC handling and CVC dressing. CLABSI rate declined from 4.00 (95% confidence interval (CI): 1.94-6.06) to 1.81 (0.46-3.17) per 1,000 CVC-days in P2 with an incidence rate ratio (IRR) of 0.49 (0.24-0.98, *p* = 0.043). A better response was observed in hospital A where the nurse participation at the monthly meeting was significantly higher than in hospital B (*p* < 0.001) as the percentage of feedbacks reports posted in ICU (*p* < 0.001). The decline in the CLABSI rate observed during P2 in comparison with P1 was independent of the insertion site (femoral or non-femoral; *p* = 0.054). The overall CLABSI rate increased to 2.73 (1.17-4.29) per 1,000 CVC-days with IRR of 0.67 (0.36-1.26, *p* = 0.212) in P3 compared to P1, but a high nursing turnover was observed in both hospitals.

**Conclusions:**

Our intervention focused on external auditing and performance feedback resulted in significant reduction in rates of CLABSI. Investigation continues regarding the most effective way to sustain CLABSI prevention practices and to improve the culture of safety in healthcare.

## Background

Bloodstream infections associated with the insertion and maintenance of central venous catheters (CVC) are the most common cause of health care-associated infections in intensive-care units (ICU) [1] and result in significant morbidity, prolonged length of stay, and excess healthcare costs [2].

Prevention of central line-associated bloodstream infection (CLABSI) remains a major issue for patient safety and costs. In fact, the CLABSI rate is proposed as an indicator of quality of care in ICU in several countries. Studies have shown that education and training of ICU health care workers (HCW) concerning CVC care is efficient in preventing CLABSI. Interventions focusing primarily on central-line insertion procedures, emphasized staff education, compliance to basic hygiene, and timely removal of CVC have been associated with substantial reductions in CLABSI rates [3-9]. Few studies have included the evaluation of post-insertion care [10-13].

Moreover, the prevention of CLABSI is a team effort involving all categories of HCW. Providing feedback on surveillance data to ICU staff has been associated with a reduced rate of hospital-acquired infections [14]. Such feedback is a useful complement to other strategies particularly feedback regarding adherence to good practices of CVC care [8,15,16]. The published CLABSI guidelines provide recommendations for implementing a checklist to ensure compliance with evidence-based practices and for empowering nurses to ensure compliance with the checklist (self-report method) [17].

The aim of this prospective interventional study was to measure the added effectiveness of monitoring by an external infection control team and continuous feedback regarding performance indicators to decrease CLABSI rates. The secondary objective was to evaluate the sustainability of the project when discontinuing monitoring.

## Methods

### Study design

This multicenter quasi-experimental study was conducted in five adult ICUs, designated ICU A1 (12 beds), A2 (12 beds), A3 (9 beds), B1 (10 beds) and B2 (10 beds), located in two tertiary hospitals A (853 beds) and B (509 beds) in Brussels, Belgium. Each ICU had mixed (medical and surgical) intensive-care beds with a separate ICU team (nurses and physicians). Before the beginning of the study, no surveillance data on the CLABSI rate or systematic recording of CVC-days was performed.

### Study phase

The study was performed from March 2011 to September 2012 in 3 phases: a pre-intervention phase (Phase (P) 1), from March 2011 to August 2011, an intervention phase (P2), from September 2011 to February 2012 and a post-intervention phase (P3), from March 2012 to September 2012.

### Study setting

Written policies and procedures that incorporated all aspects of the CVC care, maintenance and insertion processes were available to ICU staff. CVC kits that contained all the supplies necessary to comply with the sterile procedure for CVC placement were available in each ICU. Lines were placed by attending physicians using an aseptic technique and avoiding the femoral vein for CVC insertion. The local procedures for CVC insertion followed standard guidelines, i.e., use of maximal barrier precautions, skin antisepsis and meticulous hand hygiene. Skin antisepsis was performed with 0.5% chlorhexidine in 70% alcohol (Cedium®, QUALIPHAR) or with 5% alcoholic povidone-iodine (Iso-Betadine® solution hydroalcoolique, MEDA Pharma). The same types of material were used in each ICU. In particular, antimicrobial coated catheters, antiseptic-impregnated catheters, chlorhexidine dressings and patches were not used.

Two meetings of one hour per ICU were organized in August 2011 to explain the study and remind the staff of the evidence-based procedures known to decrease rates of CLABSI. One meeting was held for medical and nurse ICU heads, and one meeting was held for ICU staff. The objectives proposed to the ICU staff were zero femoral site use, at least 80% adherence with CVC-care recommendations and a 50% decline in the CLABSI rate.

Throughout the study phase, the medical record of every patient in the ICU for at least 48 hours and with positive blood cultures was reviewed by each local infection control team using a common data collection form. All positive blood cultures episodes were thereafter subcategorized locally as CLABSI, primary bloodstream infection (BSI) (no CVC), secondary BSI, or contaminant. Each case was reviewed by the investigator team.

The prevention program is fully described in Table 1. During the intervention phase, no new CVC care procedure was modified or implemented. Observations of adherence with recommendations for CVC management were performed randomly, usually during the higher-activity phase of the day (offering the most opportunities to observe), once a week during 45–60 minutes in each ICU. This external monitoring was assessed by the same trained research nurse for all of P2. In addition, The ICU staffs were aware that compliance with CVC insertion and maintenance practices was recorded. During audits of CVC care, the ICU nurses were encouraged to modify their practice when it appeared that their clinical practice was inconsistent with accepted guidelines. During a monthly meeting of approximately 20–30 minutes in each ICU, reported performances were presented to support nurse staff efforts and to promote accountability. Moreover, a monthly updated feedback report was posted in each ICU by the study investigator. Variations in nurse staffing, including the percentage of staff turnover and percentage of pool nurses were recorded. Data on the ICU physicians and nurses’ participation at the monthly meetings organized during P2 and the percentage of feedback posted by the ICU staff throughout the ICU during P3 were also collected.

**Table 1:** Description of the central line infection control program

	**Baseline**	**Phase 1 (March 2011 -August 2011)**	**Phase 2 (September 2011-February 2012)**	**Phase 3 (March 2012 -September 2012)**
**Care process**	1. Appropriate hand hygiene before and after any CVC care	As baseline	As baseline	As baseline
	2. Use of maximal barrier precautions and skin antisepsis (0.5% chlorhexidine in 70% alcohol or alcoholic povidone-iodine) before CVC insertion			
	3. Replacement of gauze dressing every 24 hours or when damp, loose, or visibly soiled replacement of transparent dressings every 7 days or when damp, loose, or visibly soiled			
	4. Disinfection of catheter hubs and injection ports before they are accessed with an appropriate antiseptic (chlorhexidine in 70% alcohol or 70% alcohol)			
	5. Traceability of information about CVC, dressing and lines (dating of placement)			
**Monitoring of outcome measures**	None	CLABSI/1,000 CVC-days, CVC utilization ratio	CLABSI/1,000 CVC-days, CVC utilization ratio	CLABSI/1,000 CVC-days, CVC utilization ratio
**Monitoring of process measures**				
Type of insertion site	None	Point prevalence survey of type of insertion site (internal jugular, subclavian, or femoral veins)	Point prevalence survey of type of insertion site as in phase 1	Point prevalence survey of type of insertion site as in phase 1
Care process	None	None	Monitoring of 5 CVC care process. Compliance rate with each care process was calculated by dividing the number of actions performed by the number of appropriate actions, expressed as a percentage	None
**Staff meetings**	Two meetings of study information and staff education per ICU	None	Monthly meeting with ICU staff to report outcome and process indicators	None
**Feedback reports**	None	None	Monthly feedback reports of outcome and process indicators posted in each ICU by the study investigator	Monthly feedback of outcome indicators sent via e-mail to ICU leaders Feedback posted in the ICU at the ICU leaders’ discretion

### Definitions

We used the definitions for BSIs of the Belgian surveillance protocol corresponding to the 1998 CDC National Nosocomial Infections Surveillance guidelines [18]. CLABSI was defined as primary BSI with a central venous catheter present for the last 48 hours. Secondary bacteremia was defined as BSI that developed as the result of a documented infection with the same microorganism at another site. The incidence density rate was defined as number of CLABSI cases per 1,000 CVC-days. For each phase, the total CLABSI incidence density rate was calculated as the mean of monthly CLABSI incidence density rates. The CVC-utilization ratio was obtained by dividing the number of CVC-days by the number of patient-days. If a patient had more than one CVC, only one was counted.

Participation in feedback meetings was expressed for nurses as a percentage (number of nurses present at each meeting compared to the number of nurses in the ICU in this phase) and for ICU physicians as a dichotomous variable (participation was considered as effective if at least 2 physicians were present at each meeting).

Regular staff was defined as nurses permanently assigned to the ICU (expressed in full-time equivalents). Pool staff was defined as nursing staff who were employees of nursing agencies. Pool staff was assigned to the ICU for different lengths of time. The staff turnover was defined as the percentage of regular ICU nurses leaving the ICU between the beginning and the end of the study.

The rate of feedback displayed during P3 was defined as the number of posters displayed in the ICU over the number of feedback reports addressed by the investigator team.

### Statistical methods

The results are expressed as the median (interquartile range) or as the mean (SD), as appropriate. Categorical variables were assessed with a Chi-square test or Fisher’s exact test when applicable. A Cochran-Mantel-Haenszel test was used in the analysis of stratified categorical data. As used in previous studies [9], and because CLABSI are rare events, a Poisson regression analysis was used to generate an incidence-rate ratio (IRR) compared with pre-intervention CLABSI incidence density rates. One-way ANOVA test was applied to evaluate statistically significant difference between the CVC-utilization ratios across the different phases.

A 2-sided *p-*value *<* 0.05 was considered statistically significant. All data analyses were performed using Stata software, version 12.0 (StataCorp LP, College Station, Texas, USA).

### Ethical considerations

The institutional review boards at each hospital approved the study protocol (number CE 2011/31 for Brugmann University Hospital and number AK/11-03-31/4011 for Saint Pierre University Hospital).

## Results

During the three study phases, there were 18,467 CVC-days. There was no statistically significant difference of the CVC-utilization ratio between the three phases (p = 0.679, one-way ANOVA test) (Table 2).

**Table 2:** Primary BSI, Secondary BSI and CLABSI rates per each phase

**Phase**	**Admissions**	**Patient-days**	**CVC-days**	**Mean CVC-utilization ratio**	**Total BSI episodes**	**Primary BSI (no CVC)**	**Secondary BSI (per 1,000 Patient- days)**	CLABSI [mean CLABSI per 1,000 CVC-days*]
1	1,354	7,604	6,045	0.79 ± 0.07	37	1	12 (1. 58)	24 [4.00 (1.94-6.06)]
2	1,571	7,882	6,399	0.80 ± 0.11	33	6	15 (1.90)	12 [1.81 (0.46-3.17)]
3	1,439	7,615	6,023	0.78 ± 0.10	44	0	28 (3.68)	16 [2.73 (1.17-4.29)]
Total	4,364	23,101	18,467	0.79 ± 0.09	114	7	55 (2.38)	52 [2.85 (1.97-3.72)]

*Incidence Rate Ratio (95% Confidence Interval) and *p*-value (Poisson regression).

In the phase 2 compared to phase 1: 0.49 (0.24-0.98), *p* = 0.043.

In the phase 3 compared to phase 1: 0.67 (0.36-1.26), *p* = 0.212.

In the phase 3 compared to phase 2: 1.37 (0.65-2.89), *p* = 0.413.

### Process indicators

**Figure 1:**
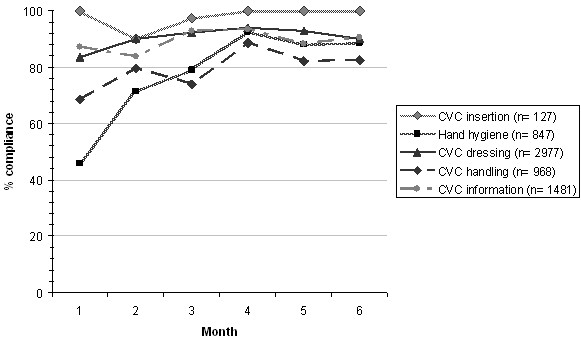
Compliance of CVC care recommendations.

Monitoring of process measures was performed during a total of 111 hours. The rates of compliance with recommendations for CVC insertion and maintenance are represented in Figure 1. Compliance with hand hygiene was 46% in the first month and increased significantly during months 2 to 6 of P2 (Relative risk [RR], 1.80; 95% confidence interval [CI]: 1.52-2.13, *p* < 0.001). Compliance with CVC handling was 69% in month 1 and also increased significantly (RR, 1.17; 95% CI: 1.03-1.33, *p =* 0.003) as did compliance with CVC dressing (RR, 1.10; 95% CI: 1.05-1.15, *p* < 0.001). The median percentage of compliance during the 6 months of the interventional phase (interquartile range) with recommendations for adequate CVC care was distributed as follows: CVC insertion 100 (90–100), hand hygiene 84 (46–93), CVC dressing 91 (83–94), CVC handling 81 (69–89), and notification of CVC and perfusions 90 (84–94). The median compliances for CVC care were not significantly different between hospitals A and B.

### Microbiological features

In P1, 69% of microorganisms identified were Gram-positive, followed by Gram-negative organisms (27%) and fungi (4%). In P3, the same proportion of microorganisms was Gram-positive (69%), Gram negative (25%) and fungi (6%). In P2, 56% of microorganisms identified were Gram-negative, followed by Gram-positive organisms (38%) and fungi (6%). Most common organisms identified in both three phases were *Streptococcus species* and *Enterococcus species* accounted for over 50% of the Gram-positive pathogens.

### Outcomes indicators

During P2, a significant reduction in the overall CLABSI incidence density rate was observed with an incidence rate-ratio (IRR) of 0.49 (*p* = 0.043), and no change in the rate of secondary bacteremias. There was a 32% CLABSI incidence density rate reduction between P3 and P1, but the difference was not significant with IRR of 0.67 (*p* = 0.212) (Table 2).

During P1, the CLABSI rates were 3.63 (1.47-5.79) and 4.54 (0.23-8.85) per 1,000 CVC-days in hospitals A and B, respectively. Both hospitals presented reductions in the CLABSI incidence density rates during P2 in comparison with P1:1.34 (0.10-2.59) with IRR of 0.38 (0.13-1.05, *p* = 0.063) and 2.32 (0.04-4.68) with IRR of 0.63 (0.24-1.62, *p* = 0.333) in hospitals A and B, respectively. During P3 in comparison with P2, the CLABSI incidence density rate increased to 2.83 (1.32-4.35) in hospital A, with IRR of 1.99 (0.68-5.81, *p* = 0.210) and remained stable at 2.74 (0.58-6.05) in hospital B with IRR of 0.92 (0.31-2.73, *p* = 0.876).

The rate of femoral CVC-use decreased between P2 (11%) and P1 (22%; RR, 0.50; 95% CI: 0.31-0.83, *p* = 0.01) and later increased between P3 (20%) and P2 (RR, 1.74; 95% CI: 1.06-2.87, *p* = 0.03). The numbers of femoral CLABSI among the total estimated femoral CVC-days were 7/1330, 3/704 and 3/1205 during P1, P2 and P3, respectively. The decline in the CLABSI rate observed during P2 in comparison with P1 was independent of the insertion site (femoral or non-femoral; Cochran-Mantel-Haenszel test, *p* = 0.054).

### Organizational indicators

Comparative organizational parameters between hospitals A and B are reported in Table 3. The hospital A differs significantly from hospital B by a higher variation in nurses staffing, a higher nurses’ participation in the monthly meetings and a higher percentage of feedbacks reports posted in ICU during P3.

**Table 3:** Comparative analysis of organizational indicators between hospital A and hospital B

	**Hospital A**	**Hospital B**	***p-*** **value**
Pool nurses	110 (13%)	41 (8%)	0.005
Staff nurse turnover	26%	9%	0.025
Nurse participation	115 (88%)	62 (61%)	<0.001
Number of meetings with the expected physicians participation	13 (72%)	5 (42%)	0.136
Number of feedback reports posted	16 (89%)	0 (0%)	<0.001

## Discussion

The objective of this study was to examine the effect on CLABSI rate of an external monitoring of CVC care compliance process with feedback aimed mainly at nurses. The major role of ICU nurses in reducing CLABSI rates was previously demonstrated with education programs dedicated especially to nurses [4,5]. To optimize prevention, it is now accepted that the “bundle” concept should be implemented, including five simple interventions supported by strong scientific evidence for effectiveness: optimal hand hygiene, chlorhexidine skin antisepsis, maximal barrier precautions for CVC insertion, use of optimal insertion sites, and prompt catheter removal [17]. This bundle is currently being used in several countries. Numerous studies have reported successful results after implementation of a continuous, multifaceted strategy based on these measures [3-8,19]. In our study, compliance with insertion recommendations (observed in the first month of P2) was initially high, most likely because of previous sensitization and use of CVC kits. More recent studies suggest an added benefit from broadening prevention strategies to include evidence-based best practices for central-line maintenance [10,11]. We chose to also monitor post-insertion CVC care such as quality of dressing, systematic disinfection before use of any access points (hubs-connectors-injection ports) and hand hygiene before CVC contact. Our intervention resulted in a transient but significant reduction in CLABSI rates and the median percentage of compliance with each procedure for CVC care was greater than 80%. As demonstrated by Furuya et al., the use of a CVC bundle is associated with lower infection rates only when compliance is high [20]. On the other hand, we observed no decrease in the CVC utilization ratio, but we did not implement a program focused on daily review of unnecessary catheters except indirectly with the surveillance of CVC placement dating. Finally, we achieved a significant decrease in femoral access during P2, with limited effect on the CLABSI rate. It is known that the risk of CLABSI varied according to the site of central venous access [21]. But we compared only femoral central venous access site to non-femoral central venous access site without regarding in details subclavian and internal jugular routes.

The difference of CLABSI incidence rate reduction observed between P2 and P1 in hospitals A and B could be explained by the significantly different participation of nurses recorded in the monthly meetings, reflecting less engagement at hospital B. We also observed a lack of investment by the ICU physicians as represented by their absence from the monthly meetings and the small amount of feedback posted in the ICU during P3 in hospital B. This may have influenced the outcome, as the HCW in the last may be less professionally motivated than the others. Ensuring staff understand the change process is fundamental to the success of a quality improvement.

We observed a non significant reduction of CLABSI incidence rate in P3 in comparison with P1, while Pronovost et al. [9] reported a sustained reduction of up to 66% in CLABSI rates after 18 months of follow-up in a larger study with an educational intervention based mainly on use of the CVC insertion bundle. Potentially, the lack of individual face-to-face contact and feedback maybe explains the lack of sustainability of our program, as described in another study [22]. In that study, each HCW was approached individually regarding the insertion technique and maintenance of CVC and therefore felt valued and motivated. Our approach involved collective feedback through monthly and non-mandatory meetings. Successful practice changes require buy-in from participants. Therefore, it is necessary to pay close attention to perceived barriers. More research is needed to develop a more direct measure of HCW engagement in quality and safety.

Another explanation for the declining performance during P3 could be the high turnover in nurse staffing and the high percentage of pool nurses recorded, particularly in hospital A. Robert et al. suggested in a case–control study that nurse staffing composition (i.e., pool-nurse–to-patient ratio) might be related to primary BSI risk [23]. In our study, the monitoring of process indicators was terminated at the end of P2, and in the following months, there were no more clinical reminders for ICU staff. Moreover, no specific training related to line care was planned for pool staff. Based on our experience, account should be taken of these structural parameters to adjust in further studies the frequency of process measurements. Finally, feedback alone (as done in P3) was ineffective for altering provider behaviors.

There are a number of limitations to this study. First, it is not a randomized trial, but a quasi-experimental study without a concurrent control group, a design frequently used for this type of study. Thus, other unmeasured factors, such as long-term trends or seasonal confounders might have occurred coincident with the intervention resulting in independent variations in CLABSI rates [24]. Second, unmeasured factors such as patient risk severity may have confounded CLABSI rates; however, it is unlikely that the case composition would have changed in the same manner during the intervention phase in all 5 ICUs.

## Conclusions

Our encouraging results emphasized the value of this intervention based on auditing and feedback to reinforce practice changes to decrease CLABSI rates. This intervention was dependant on local factors such as the lack of leadership and support inside the ICU and the high turnover of ICU nurses, as illustrated by the difference observed between the 2 hospitals despite a targeted compliance with CVC care recommendations achieved. Our study underlines the need to monitor behavioral parameters in addition to process and results indicators, to analyze the benefits of any new infection control program. Further studies are needed to determine which strategies are most effective in changing professional behavior and in promoting long-term, sustained adherence to evidence-based practices for CLABSI prevention.

## Competing interests

The authors declare that they have no competing interests.

## Authors’ contributions

SC and BB contributed to the conception of the study. SC, SA and MG participated in the data collection. SA and BB contributed to statistics. SC, SA, MG and BB interpretated data. All authors read and approved the final manuscript.
